# A mosquito feeding assay to examine *Plasmodium* transmission to mosquitoes using small blood volumes in 3D printed nano-feeders

**DOI:** 10.1186/s13071-020-04269-x

**Published:** 2020-08-08

**Authors:** Wouter Graumans, Roel Heutink, Geert-Jan van Gemert, Marga van de Vegte-Bolmer, Teun Bousema, Katharine A. Collins

**Affiliations:** 1 Department of Medical Microbiology, Radboud University Medical Center, Radboud Institute for Health Sciences, Nijmegen, The Netherlands; 2 Department of Immunology and Infection, London School of Hygiene and Tropical Medicine, London, UK

**Keywords:** Malaria, Transmission, DMFA, SMFA, *Anopheles*, Blood-meal size, 3D printing, Feeders, Temperature, Oocyst

## Abstract

**Background:**

To understand the dynamics of malaria transmission, membrane feeding assays with glass feeders are used to assess the transmission potential of malaria infected individuals to mosquitoes. However, in some circumstances, use of these assays is hindered by both the blood volume requirement and the availability of fragile, specially crafted glass feeders. 3D printed plastic feeders that require very small volumes of blood would thus expand the utility of membrane feeding assays.

**Methods:**

Using two 3D printing production methods, MultiJet (MJ) and Digital Light Processing (DLP), we developed a plastic version of the most commonly used standard glass feeder (the mini-feeder) with an improved design, and also a smaller feeder requiring only 60 µl of blood (the nano-feeder). Performance of the 3D printed feeders was compared to standard glass mini-feeders by assessing infectivity of gametocytes to mosquitoes in standard membrane feeding assays with laboratory reared *Anopheles stephensi* mosquitoes and cultured *Plasmodium falciparum* gametocytes. In addition, the optimum number of mosquitoes that can feed on the nano-feeder was determined by evaluating fully fed mosquitoes visually and by assessing blood- meal volume with a colorimetric haemoglobin assay.

**Results:**

The 3D printing methods allowed quick and inexpensive production of durable feeders. Infectivity of gametocytes to mosquitoes was comparable for MJ and DLP 3D printed feeders and glass feeders, and the performance of the 3D printed feeders was not influenced by repeated washing with bleach. There was no loss in transmission efficiency when the feeder size was reduced from mini-feeder to nano-feeder, and blood-meal volume assessment indicated ~10 *An. stephensi* mosquitoes can take a full blood-meal (median volume 3.44 µl) on a nano-feeder.

**Conclusions:**

Here we present 3D printed mini- and nano-feeders with comparable performance to the currently used glass mini-feeders. These feeders do not require specialized glass craftsmanship, making them easily accessible. Moreover, the smaller nano-feeders will enable evaluation of smaller blood volumes that can be collected from finger prick, thus expanding the utility of membrane feeding assays and facilitating a more thorough evaluation of the human infectious reservoir for malaria.
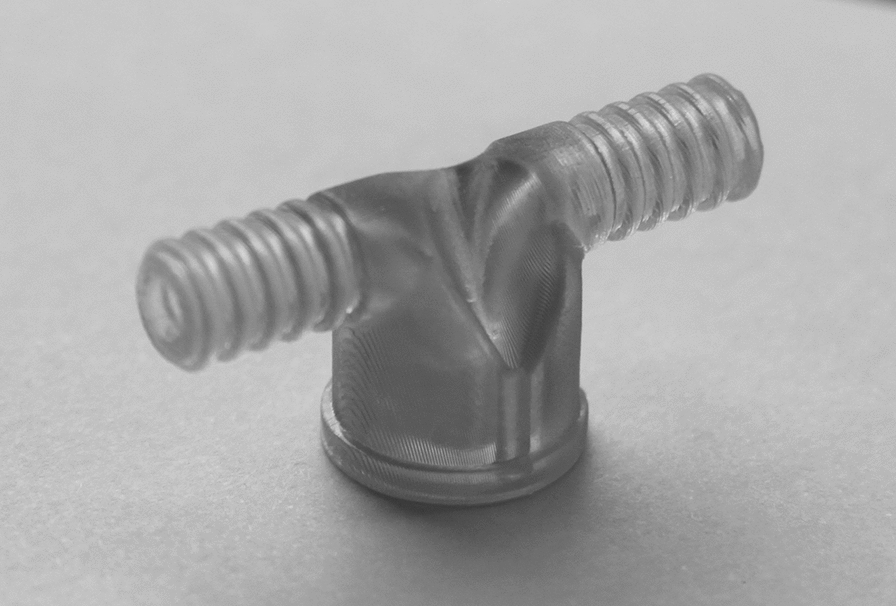

## Background

Malaria remains one of the most important infectious diseases worldwide with an estimated 228 million cases and 405,000 deaths occurring in 2018 [1]. Despite reductions in morbidity and mortality during the last decade, the number of cases globally has now plateaued and is even rising again in some settings. In particular children under the age of five in the African region are most affected, with (severe) morbidity largely attributable to infection with *Plasmodium falciparum*. Efforts to further reduce malaria burden would benefit from an improved understanding of malaria transmission dynamics [2]. The likelihood of malaria transmission from humans to mosquitoes is dependent on the presence of mature gametocytes. Gametocytes are the sexual forms of the malaria parasite, and for *P. falciparum* they enter the peripheral blood stream after ~10–12 days of maturation and circulate for an estimated 3.4–6.5 days [3–5]. Gametocyte infectivity to mosquitoes depends on numerous factors and cannot be reliably inferred from gametocyte density alone [6, 7].

Artificial mosquito membrane feeding assays (MFAs) are widely used to evaluate the transmission potential of gametocyte-infected blood to mosquitoes. These assays are used largely for two reasons: (i) to assess the efficacy of interventions that aim to reduce transmission of malaria parasites from humans to mosquitoes; and (ii) to evaluate malaria transmission dynamics and identify the transmissible reservoir of malaria [6, 8–12]. During an MFA gametocyte infected blood is fed to mosquitoes *via* a membrane feeding device connected to a circulating water bath to keep the blood warm and prevent premature activation of gametocytes by a drop in temperature [13]. Transmission of gametocytes to mosquitoes is then assessed by detecting and quantifying malaria oocysts on mosquito midguts 7–10 days after feeding [14]. Water-jacketed glass membrane feeders were initially developed by Rutledge et al. [15] in 1964 to facilitate MFAs. In 1989, the design was improved to produce glass feeders in three sizes for blood volume capacities of 8.0 ml, 1.25 ml, and 0.3 ml [14], called maxi-, midi-, and mini-feeders, respectively. Glass feeders have been successfully used in many settings; however, they are expensive, and standardized feeders can only be obtained from a few suppliers, limiting their availability to all laboratories. 3D printing technology could be used to produce feeders more easily and inexpensively, thus making them more attainable by laboratories with limited resources. A 3D printed feeder model using 500 µl of blood was previously produced where the feeders were printed using acrylic resin photopolymer in two pieces that were glued together after production [16].

Despite the specialized nature of the manufacturing process, glass midi- and mini-feeders have been widely used for clinical and epidemiological studies on human infectivity to mosquitoes using direct membrane feeding assays (DMFAs) [12, 17]. In DMFAs, venous blood collected from gametocyte carriers is added directly to a glass feeder and fed to mosquitoes to assess infectivity. Performing DMFAs on populations where phlebotomy is challenging, such as infants [18], or using methods to concentrate gametocytes prior to mosquito feeding [19–21] would benefit from feeders using smaller blood volumes, such as those that could easily be collected by finger prick. For these purposes, we have developed the nano-feeder with a chamber volume of only 60 µl. Utilising recent advances in 3D printing technology, we produced the mini- and nano-feeders in plastic, in one piece, with an improved design. Here we present the validation of these feeders using standard membrane feeding assays (SMFAs) which use cultured *P. falciparum* gametocytes and *Anopheles stephensi* mosquitoes.

## Methods

### Nano-feeder development and 3D printing technology

Two 3D printing methods were used: MultiJet (MJ) and Digital Light Processing (DLP). Feeders were designed using 3D CAD software for MJ printed feeders (SolidWorks 2019 SP0.0, Dassault Systèmes, ‘s-Hertogenbosch, The Netherlands) and 3DSprint software for DLP printed feeders (3Dsystems, Rock Hill, USA), and were printed in one piece (STL files for the feeders are provided in Additional files 1 and 2). The MJ printing process was performed by the 3D Projet 3500 max printer by the Civon Innovation Center. Briefly, feeders were printed with ABS like plastic (VisiJet M3-X, 3Dsystems, Rock Hill, USA) and wax (VisiJet S300, 3Dsystems, Rock Hill, USA) was printed as temporary support material for the layers printed on top. Post-printing wax was removed in a multistep process at 65 °C, feeders were heated in the oven, ultrasonic cleaned in oil, washed with water, cleaned with a brush and dried with compressed air. The DLP printing process was performed by the Nextdent 5100 Beta printer by the Radboudumc 3D laboratory (Additional file 3: Figure S1) [22]. Nextdent SG (surgical guide) was used as resin and no support material was needed. Each layer of printed liquid resin was hardened by UV beamer projection (xyz resolution 50 µm). Post-printing, feeders were ultrasonically cleaned in 96% ethanol twice, then flushed with 96% ethanol, dried with compressed air, and post-cured for 10 minutes in a UV box (Nextdent LC-3DPrint Box, UV-A 108 en UV-Blue 108, 3Dsystems). Glass mini-feeders and 3D printed nano-feeders were connected to a circulating water bath (temperature set to 39 °C) and blood was added to the feeders. A calibrated digital thermometer (54II, Fluke, Everett, USA) with an accuracy of ± 0.3 °C was used with two probes to measure simultaneously the temperature of the blood in the feeder cavity of the first and last feeder in a row of four, every 30 s for 10 min, to determine temperature stability.

### *Plasmodium falciparum* gametocyte culture, *Anopheles stephensi* husbandry and membrane feeding assays

*Plasmodium falciparum* parasites were cultured in an automated incubator [23] under a continuous gas flow of 4% CO_2_, 3% O_2_ and 93% N_2_. For gametocyte production, asynchronous cultures were started at day 0, with 1% parasitemia and 5% red blood cells, and harvested at day 15–16. Gametocyte infected blood-meals were prepared as previously described [14]. *Anopheles stephensi* mosquitoes of the Sind-Kasur strain [24] were maintained at 26 °C and 80% humidity with a 12:12 h reverse day and night cycle. For feeding assays, female mosquitoes between 1–3 days-old were offered a *P. falciparum* gametocyte infected blood-meal in either (i) paper cups (8.5 × 11 cm, 400 ml) *via* mini-feeders (300 µl) [14] or (ii) plastic cages with netting on both sides (5 × 5 × 7 cm) or small paper cups (6.5 × 8 cm, 200 ml) *via* nano-feeders (60 µl). Feeders were attached to a circulating water bath set at 39 °C. Parafilm M was stretched as artificial membrane and secured around the feeder by an elastic band. Mosquitoes were allowed to feed in the dark for 15 min and then either dissected on the same day as feeding for blood-meal haemoglobin analysis (detailed below) or maintained at 26 °C, on 5% glucose and dissected 7–10 days post-feeding to count the number of developed oocysts by microscopy after 1% mercurochrome midgut staining.

### Standard feeder washing procedure

Feeders were cleaned for 30 min in 4% bleach, rinsed with continuously running tap water for 1 h on the outside, attached to tubing to rinse the inside with running tap water for 1 h, then rinsed on the outside for another 30 min. Finally the feeders were rinsed with deionized water and air-dried.

### Method of dissection for mosquito blood-meal volume analysis

Mosquitoes were fed freshly drawn venous blood collected in EDTA vacutainers (BD, Vacutainer system) by glass mini-feeders to obtain fully blood-fed (FBF) and partially blood-fed (PBF) mosquitoes. Unfed mosquitoes (UF) were collected from the same mosquito stock cages but were not offered a blood-meal. Mosquitoes were dissected either immediately or 3 h (± 0.5 h) after feeding by separating abdomens from the rest of the body (legs, thorax and head). Blood-meal volume was determined in the abdomens or they were processed further to obtain midguts for blood-meal volume analysis. The mosquito blood-meal volume was measured using the Haemoglobin Colorimetric Assay Kit (Cayman, 700540, Ann Arbor, USA). Individual abdomens or midguts collected as described above, were transferred to a well of a 96-well plate containing 20 µl kit sample buffer. Two standard curves were prepared; the haemoglobin assay kit curve and blood donor curve. The latter was used to correlate OD against blood volume, since haemoglobin concentrations differ between donors. One hundred and eighty µl of haemoglobin detector was added to all wells. Samples were gently but thoroughly pipetted up and down to release the blood. For assessments on the abdomen, residual undissolved parts of the abdomen were removed with a needle. After 15 min of incubation, absorbance was measured by a micro plate reader at 580 nm. Donor-specific blood standard curves were used to calculate engorged mosquito blood-meal volumes.

### Statistical analysis

Data were analysed with Graphpad Prism version 8. The D’Agostino-Pearson normality test was used to determine if the data were normally distributed. Three groups of parametric data were compared by one-way ANOVA with Tukey’s multiple comparison test and two groups of non-parametric data were compared by Mann-Whitney test. Significance was indicated when value of *P* < 0.05 (*P < 0.05, **P < 0.01, ***P < 0.001).

## Results

### 3D printed nano- and mini-feeders

**Fig. 1:**
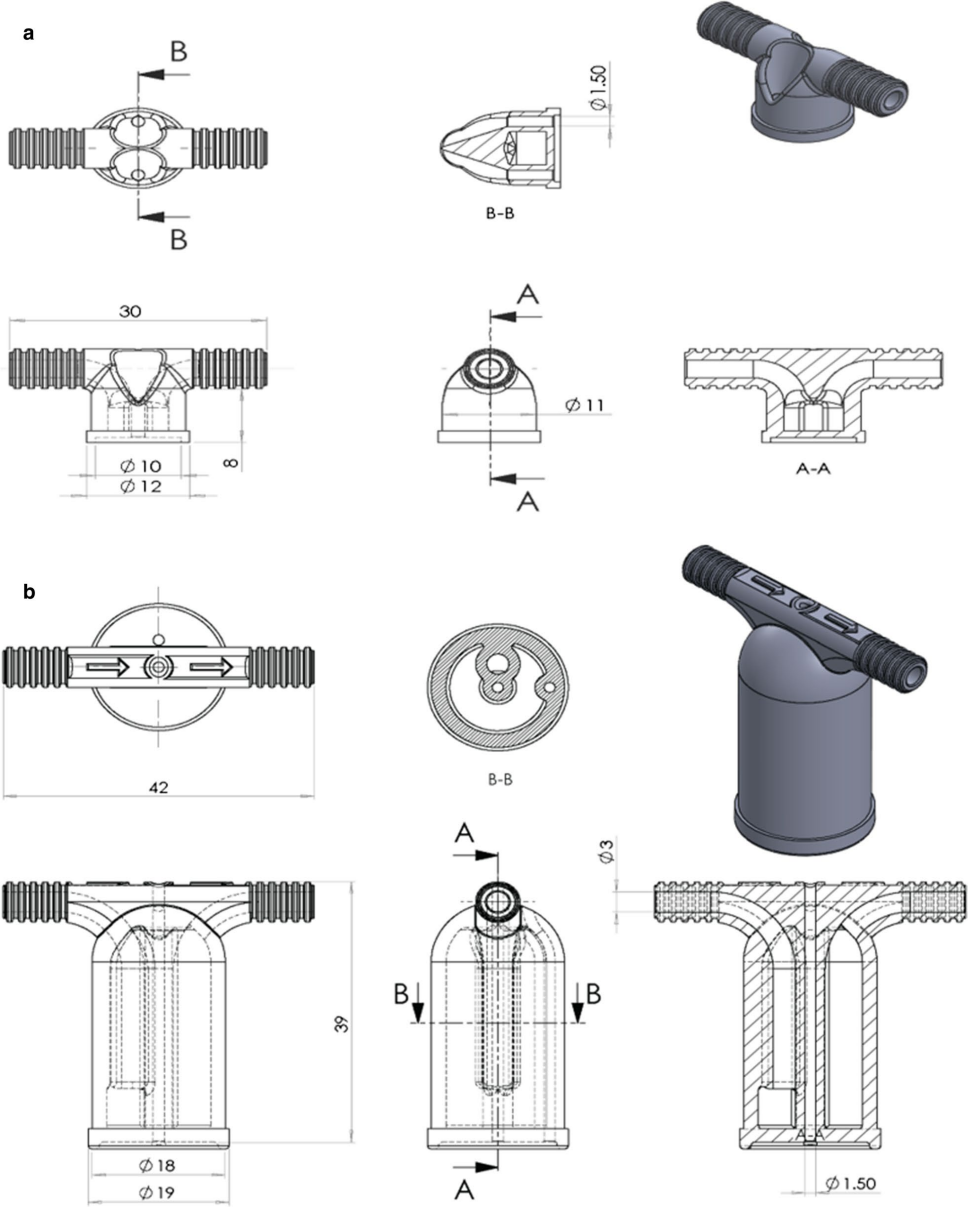
Technical design of the 3D printed nano- and mini-feeder. Nano-feeders (**a**) and mini-feeders (**b**) were 3D designed and produced by MJ and DPL 3D printing technology. The arrows labelled A and B indicate the location of the cross-sectional view of the design. The design of both feeders was improved by adding a venting tube to release air during feeder filling. For the nano-feeder the injection duct was modified to fit a p200 pipette tip to fill the feeder with blood

Nano- and mini-feeders were designed for production using MJ and DLP 3D printing technology (Fig. 1). The design of both feeders was improved by adding a venting tube to release air during feeder filling, thus enabling the chamber to be filled evenly with blood without air bubbles. Additionally, for the nano-feeder the injection duct was modified to fit a p200 pipette tip to make it easier to fill the feeder with blood and avoid the need for blunt needles [25]. After production, the MJ printed feeders had a thin layer of wax (the material that was used as temporary support layer during printing) that was difficult to remove and stayed visible on the feeders, which was not present on the DPL feeders. To assess temperature stability, the feeders were connected in a chain of 4 to a circulating water bath set at 39 °C and filled with blood. Temperature of the blood was measured in the first and last feeder using a calibrated thermometer. Both plastic and glass feeders reached a stable temperature quickly within 4.5 min after attaching to the water bath. Plastic feeders remained at a stable temperature on average 1.8 °C lower (mean = 35.9 °C) than glass feeders (mean = 37.7 °C) (Additional file 4: Figure S2).

### Mosquito infectivity is comparable with 3D printed plastic feeders (MJ and DLP printed) and glass feeders

**Fig. 2:**
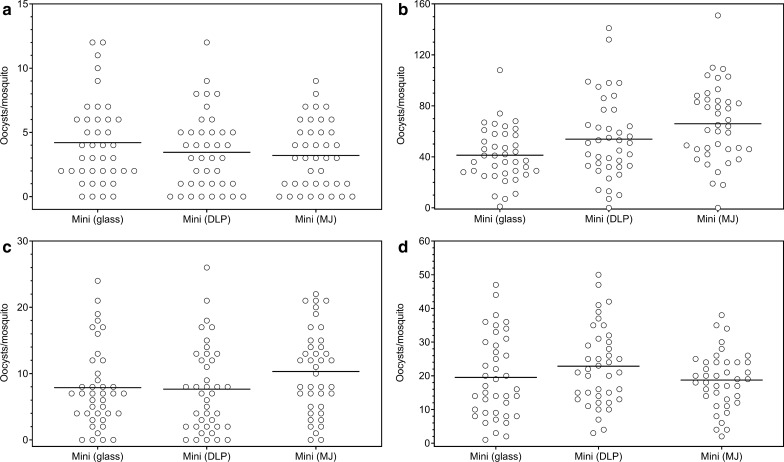
Mosquito infection rates for 3D printed feeders are comparable with glass feeders, also after repeated washing. Mosquito infectivity was assessed for MJ and DLP printed mini-feeders immediately after production (**a**) and after washing 10 times (**b**), 20 times (**c**) and 30 times (**d**) for comparability against standard glass mini-feeders. All experiments were performed with 2 paper cups of 30 mosquitoes per condition. Replicates are shown pooled, dots represent the number of oocysts per mosquito and lines represent the mean. Groups compared by one-way ANOVA with Tukey’s multiple comparison test; all were not significantly different

MJ and DLP printed mini-feeders were compared directly to glass mini-feeders in SMFAs with cultured *P. falciparum* NF54 gametocytes. To assess any potential impact of the standard Radboudumc laboratory washing procedure (using 4% bleach) on the plastic feeders, the level of mosquito infectivity was assessed immediately after production of the feeders and also after washing the feeders 10, 20 and 30 times (Fig. 2). There was a small colour change in the DLP printed plastic feeders after repeated washing with bleach, but there was no significant reduction in the mosquito infectivity compared to glass mini-feeders, either initially or following repeated washes, and there was no difference between the MJ or DPL plastic feeders. This demonstrates equal performance of the 3D printed plastic feeders compared to standard glass feeders during SMFAs and paved the way for examining smaller feeders with a smaller blood volume.

### Blood-meal volume in mosquito midguts

**Fig. 3:**
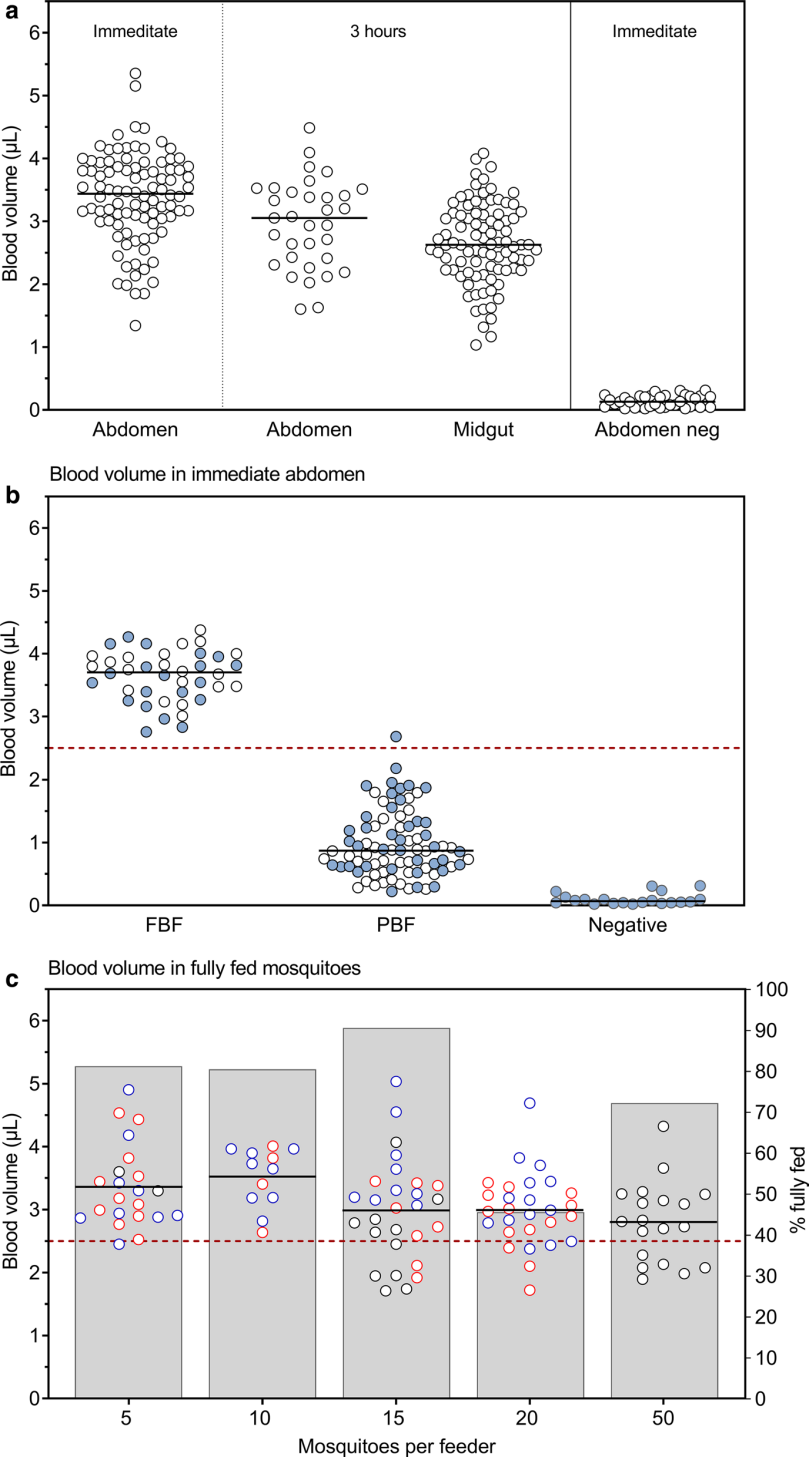
Mosquito blood-meal volume depends on the number of mosquitoes per feeder. *Anopheles stephensi* mosquitoes (30 mosquitoes per paper cup in duplicate) were fed whole blood *via* glass mini-feeders and blood-meal volumes were determined. **a** Two dissection methods (abdomen or midguts) and two time-points (immediate and 3 h ± 0.5 h after feeding) were compared. **b** Based on mosquito body size by eye (Additional file 3: Figure S2) the blood-meal volume of fully blood-fed (FBF) and partially blood-fed (PBF) mosquitoes was determined in two sets of experiments (filled and open dots). Bars represent the median. The cut-off value of FBF mosquitoes was determined to be 2.5 µl, horizontal red dotted line. **c** In three independent experiments with three different blood donors, depicted in black, blue and red, plastic cages with 5, 10, 15, 20 or 50 mosquitoes were fed on a 60 µl blood-meal *via* a nano-feeder. Fully blood-fed (FBF) mosquitoes were analysed immediately after feeding (abdomen). Data from independent experiments were pooled per group for analysis. Lines indicate the mean blood volume and bars show the prevalence of FBF mosquitoes

To evaluate how many mosquitoes can successfully feed and become fully engorged during an MFA, we wanted to determine the average blood-meal volume in FBF mosquito midguts by haemoglobin assay. Due to the difficulty of dissecting and removing a midgut immediately after feeding (engorged midguts are very delicate), three methods were evaluated to determine the optimal processing procedure for assessing the blood-meal volume: (i) whole abdomens processed immediately after feeding; (ii) whole abdomens processed 3 h (± 0.5 h) after feeding; and (iii) dissected midguts processed 3 h (± 0.5 h) after feeding. There was no difference in blood-meal volume assessed between midgut or whole abdomen processed 3 h after feeding (conditions ii and iii), indicating the additional mosquito abdomen tissue did not interfere with the assay. There was a higher median blood-meal volume in abdomens processed immediately compared to 3 h post-feeding, suggesting that some of the blood is digested by 3 h. Based on these results, we employed the approach of assessing blood-meal volumes immediately after feeding using the whole mosquito abdomens (Fig. 3a). The median blood-meal volume in whole abdomens for engorged mosquitoes was 3.44 µl (range 1.3–5.4, *n* = 94). It was noted that there was a broad range of blood-meal volumes in the mosquitoes, so we evaluated whether mosquitoes that are not fully engorged can be easily visually identified by body size (Additional file 5: Figure S3). Based on visual inspection, FBF mosquitoes had a median blood-meal volume of 3.70 µl (range 2.8–4.4, *n* = 40), considerably higher than PBF mosquitoes, median = 0.87 µl (range 0.2–2.7, *n* = 82) (Fig. 3b). An estimated mosquito blood-meal volume below 2.5 µl was therefore considered only partially fed.

### The maximum number of mosquitoes that can be fed on a nano-feeder

To determine the number of mosquitoes that can successfully feed and become fully engorged using a nano-feeder, we assessed the blood-meal volume by haemoglobin assay in mosquito midguts after feeding different numbers of mosquitoes on whole blood. In three independent experiments each with a different blood donor, cups with 5, 10, 15, 20 or 50 mosquitoes were fed on a 60 µl blood-meal *via* a nano-feeder. In all conditions, a number of mosquitoes were PBF or UF (Additional file 6: Figure S4). With increasing numbers of mosquitoes per feeder, there was a trend for lower blood-meal volume and also a larger proportion of mosquitoes with blood-meal volume < 2.5 µl, indicative of incomplete feeding (even among visually FBF mosquitoes) (Fig. 3c). If 10 mosquitoes were used per feeder, all visually FBF mosquitoes had a blood-meal volume > 2.5 µl, with a mean blood-meal volume of 3.52 µl (range 2.6–4.0, *n* = 12) (Fig. 3c).

The percentage of fully engorged mosquitoes was low in this experiment. With 10 mosquitoes per nano-feeder, 65% of the mosquitoes were FBF compared to ~ 90% with normal glass feeders. To increase mosquito feeding rates the nano-feeder procedure was optimised by: (i) extension of the starvation period prior to feed (+ 12 h); (ii) stimulation of mosquitoes prior to feeding by placing a culture flask (Corning, Canted neck 25 cm^2^) filled with warm water for 1 min on top of the cup; and (iii) plastic cages with netting on both sides were changed to small paper cups (6.5 × 8 cm, 200 ml) with netting (to reduce distance between the mosquitoes and the feeder). When 10 mosquitoes per cup were fed on the nano-feeder using the optimized protocol the mean mosquito feeding rate increased to 93.2% (range 88.9–100, *n* = 6 cups) (Additional file 7: Table S1).

### Mosquito infectivity is not affected by the smaller sized nano-feeders

**Fig. 4:**
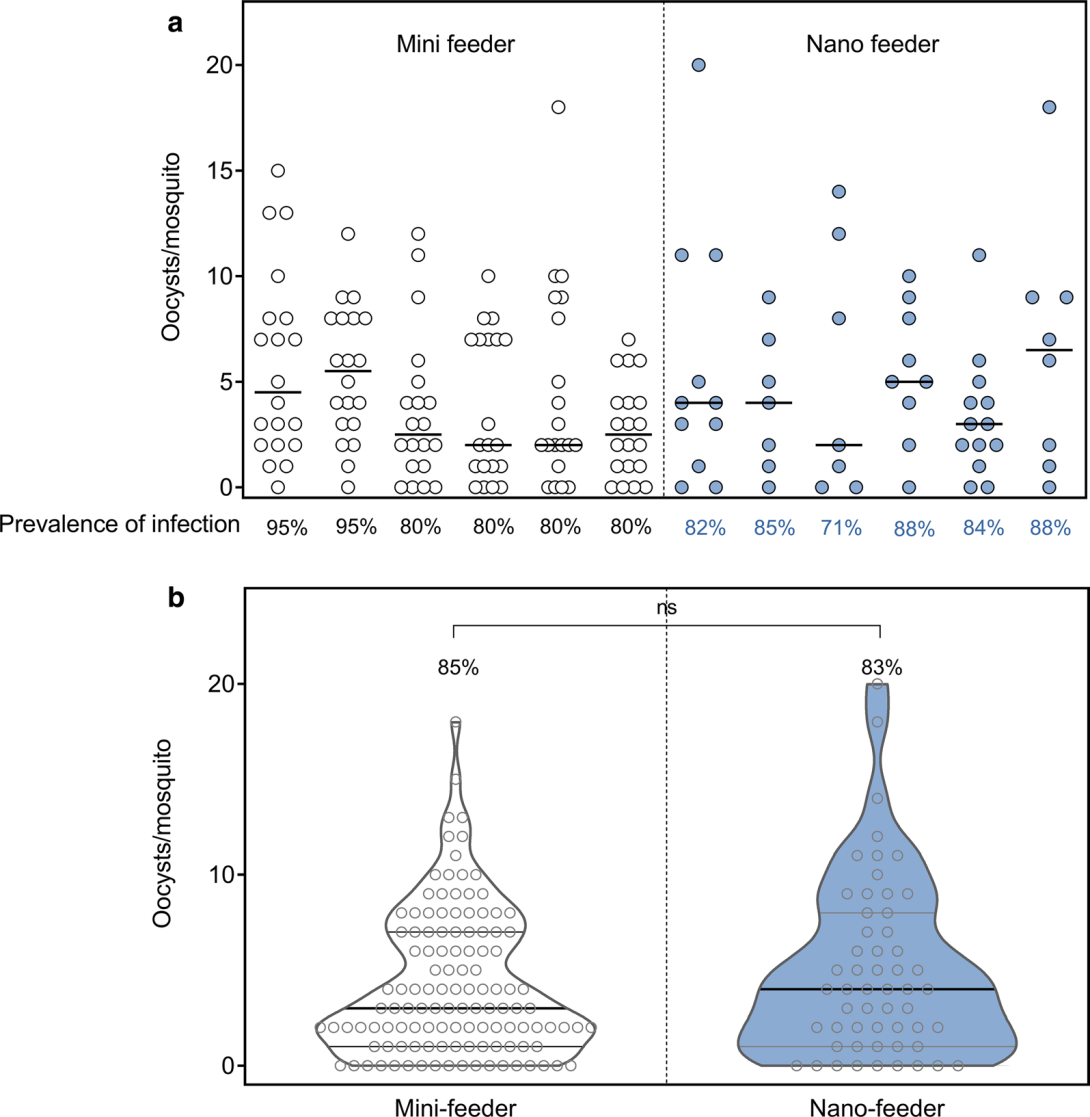
Mosquito infection intensity and prevalence are comparable between mini-feeders and nano-feeders. *Plasmodium falciparum* infected blood-meals were fed to *An. stephensi* mosquitoes in 6 glass mini-feeders (*n* = 30 mosquitoes per feeder) and 6 3D MJ printed nano-feeders (*n* = 15 mosquitoes per feeder). The infection prevalence and oocyst intensity is presented for individual feeders (**a**) (lines represent the median) and pooled by feeder type (**b**) (violin plots show the median and quartiles). Groups in **b** are compared with Mann-Whitney test, there was no significant difference

To determine if comparable mosquito infection rates can be achieved using the smaller sized 3D plastic nano-feeders, they were compared to the standard glass mini-feeders in SMFAs. Cultured gametocytes were fed to mosquitoes (six replicate feeds) and infection prevalence and intensity were compared (Fig. 4). There was no difference in median oocyst infection intensity [glass mini-feeders mean = 3.0 (range 0–18, *n* = 120) compared to 3D MJ-printed nano-feeders mean = 4.0 (range 0–20, *n* = 55)] or in prevalence of infected mosquitoes [glass mini-feeders = 85% (range 80–95) and 3D plastic feeders = 83% (range 71–88)].

## Discussion

An improved understanding of malaria transmission dynamics would enable better design and implementation of interventions that aim to reduce transmission and ultimately decrease the burden of (severe) malaria in endemic areas. In clinical and epidemiological studies, DMFAs are often performed to assess an individual’s infectivity to mosquitoes using venous blood and glass mini-feeders. However, infectivity assessments on small blood volumes for populations such as infants, where phlebotomy is challenging [18], or methods that use gametocyte concentration prior to mosquito feeding [19–21] would require feeders that use a smaller blood volume than the currently available glass mini-feeder.

In this study, plastic feeders were produced using two 3D printing techniques. 3D printing allows custom feeder optimization. We optimized feeder design by (i) adding a vent tube to allow more efficient feeder filling with blood, (ii) miniaturizing the mini-feeder design to produce a nano-feeder with a 60 µl capacity, and (iii) widening the injection duct of the nano-feeder to fit a p200 pipette tip, avoiding the need for blunt needles for feeder filling [25]. 3D printing technology was fast and inexpensive and thus may increase access to membrane feeding techniques. For example, the DLP process produced 23 nano-feeders in 90 min (Additional file 6: Figure S4), with manufacturing costs three times lower compared to current standard glass mini-feeders.

Plastic feeders were compared with the standard glass mini-feeders in SMFAs with cultured gametocytes. In these experiments, there was no difference in infection efficiency, with highly similar mean oocyst intensity and prevalence of mosquito infection. Moreover, performance of the plastic feeders was not influenced by repeated washing using bleach. These findings using two different 3D printing materials and technologies are in line with findings from Witmer et al. [16] who reported no reduction in mosquito infectivity with 3D printed feeders compared to glass feeders. For 3D printed plastic feeders it was interesting to note they remained at a stable temperature on average 1.8 °C lower (mean = 35.9 °C) than glass feeders (mean = 37.7 °C). The lower temperature could potentially influence transmission as a result of gametocyte activation before they are imbibed by the mosquitoes. However, since no reduction in mosquito infectivity was observed between the plastic and glass feeders, this lower temperature did not impact gametocyte infectivity or mosquito feeding rates, in line with previous data where gametocyte activation was only seen with a drop of 5 °C or more [13]. No difference was observed in performance between the MJ or DPL 3D printed mini-feeders, meaning both technologies appear suitable for producing feeders. However, for our purposes, DLP printed feeders were preferred because they were made out of medical certified resin resistant to autoclaving and no support material was needed during printing. In contrast, the MJ printed feeders used a wax support matrix and a thin layer of wax was present after production that was difficult to remove and remained on the feeders after cleaning.

To determine the number of mosquitoes that can be fed on a nano-feeder we measured mosquito blood-meal volumes by haemoglobinometry. In prior reports, blood-meal volumes have been estimated in a variety of mosquito species using a range of approaches, haemoglobinometry [26], IgG ELISA [27], radioisotope counting [28, 29] or simple weighing before and after blood-meal engorgement [30]. *Anopheles* mosquitoes filter and concentrate erythrocytes during feeding and excrete excess fluid, known as prediuresis [31]. Therefore, since some of the whole blood volume may be excreted, measuring heamoglobin allows us to estimate the total erythrocytes and thus the total volume of whole blood that was initially imbibed, as opposed to the total volume remaining after prediuresis. We demonstrate that blood-meal volume can be measured in whole abdomens and does not require the midgut to be removed. The highest median blood-meal volume was observed in abdomens processed immediately compared to 3 hours post-feed, likely due to erythrocyte digestion that takes place at a rate of approximately 9.8 µg of protein per hour [32] starting immediately after ingestion. Using this assay we were able to determine that the optimum number of 10 *An. stephensi* mosquitoes can be fed on a nano-feeder and take a full blood-meal (2.6–4.0 µl). Using more mosquitoes than this resulted in some mosquitoes that appeared to be FBF by visual inspection taking a smaller blood-meal as determined by colorimetric haemoglobin assay. It is possible that these mosquitoes may have appeared fully engorged because they did not fully concentrate the erythrocytes and excrete the excess fluid.

## Conclusions

In conclusion, we report an optimized robust 3D printed mini-feeder and a new smaller nano-feeder that enables gametocyte infectivity assessments on small blood volumes. 3D printed feeders showed comparable transmission efficiency compared to standard glass mini-feeders and were produced more quickly and cheaply than glass feeders, making them more widely available. These feeders expand the utility of membrane feeding assays, allowing the use of finger prick blood samples for infectivity assessments, and will facilitate a better understanding of the human infectious reservoir for malaria. Moreover, the 3D printed feeders could also facilitate studies on other important vector-borne diseases that have an impact on human health globally such as Zika, dengue, yellow fever, chikungunya and filariasis [33].

## Supplementary information

**Additional file 1.** STL file for 3D printed mini-feeders.

**Additional file 2.** STL file for 3D printed nano-feeders.

**Additional file 3: Figure S1.** DLP nano-feeder production by Nextdent 5100. DLP feeders were designed using 3DSprint software from 3Dsystems. A total of 23 nano-feeders were printed in 1 h by the Nextdent 5100 Beta printer by the Radboudumc 3D lab [22]. Nextdent SG (surgical guide) was used as resin, no support material was used. Each layer of printed liquid resin was hardened by UV beamer projection (xyz resolution 50 µm). In 30 minutes, post printing (B), feeders were two times ultrasonic cleaned in 96% ethanol and flushed with 96% ethanol before they were dried with compressed air and post-cured for 10 min in a UV box (Nextdent LC-3DPrint Box, UV-A 108 en UV-Blue 108).

**Additional file 4: Figure S2.** Feeder temperature of glass mini-feeders and 3D MJ-printed nano-feeders. Two probes were used to simultaneously measure the temperature of the first (P1) and last (P2) feeder in a row of four. At 0 s, the water bath was turned on (dotted line, temperature set at 39 °C) and the blood-meal was injected in the feeder cavity of glass mini-feeders (circles) and nano-feeders (triangles). Both plastic and glass feeders reached a stable temperature quickly within 4.5 min after attaching to the water bath. Plastic feeders remained at a stable temperature on average 1.8 °C lower (mean = 35.9 °C) than glass feeders (mean = 37.7 °C).

**Additional file 5: Figure S3.** Mosquito blood-meal size determined by eye. Mosquitoes were selected by eye and determined unfed (UF), partially blood-fed (PBF) or fully blood-fed (FBF) (from left to right).

**Additional file 6: Figure S4.** Mosquito feeding performance on 3D MJ-printed nano-feeders. In three independent experiments with three different blood donors, plastic cages with 5, 10, 15, 20 or 50 mosquitoes were fed on a 60 µl blood-meal to determine the feeding performance on the nano-feeder. Data from independent experiments were pooled per group for analysis; pie charts present the mean feeding rate in percentages of fully- (FBF), partially- (PBF) or unfed (UF) mosquitoes. In all conditions a number of mosquitoes were PBF or UF. No blood material in the feeder cavity was left for cups with 20 or 50 mosquitoes. For 10 mosquitoes per feeder, all visually FBF mosquitoes had a blood-meal > 2.5 µl, with a mean volume of 3.52 µl (range 2.6–4.0, *n* = 12) and feeding prevalence of 65% FBF.

**Additional file 7: Table S1.** Mosquito feeding rates after protocol optimization. To increase mosquito feeding rates the original nano-feeder protocol was optimised by (i) extension of the starvation period prior to feed (+ 12 h), (ii) stimulation of mosquitoes prior to feeding by placing a culture flask (canted neck 25 cm^2^) filled with warm water for 1 minute on top of cup, and (iii) plastic cages were changed to small paper cups to reduce distance between the mosquitoes and the feeder.

## Data Availability

The datasets used and/or analysed during the present study are available from the corresponding author upon reasonable request. The authors of this manuscript are willing to consider requests for printing feeders.

## Abbreviations

DLP: digital light processing
DMFA: direct membrane feeding assays
FBF: fully blood-fed
MFA: membrane feeding assay
MJ: multijet
PBF: partially blood-fed mosquitoes
SG: surgical guide
SMFA: standard membrane feeding assay
UF: unfed mosquitoes
