# Supplementary material for: A mosquito feeding assay to examine Plasmodium transmission to mosquitoes using small blood volumes in 3D printed nano-feeders
Source: Parasit Vectors. 2020 Aug 8;13:401. doi: 10.1186/s13071-020-04269-x (PMC7414548; doi:10.1186/s13071-020-04269-x)

**Additional file 3: Figure S1.** DLP nano-feeder production by Nextdent 5100. DLP feeders were designed using 3DSprint software from 3Dsystems. A total of 23 nano-feeders were printed in 1 hour by the Nextdent 5100 Beta printer by the Radboudumc 3D lab [22]. Nextdent SG (surgical guide) was used as resin, no support material was used. Each layer of printed liquid resin was hardened by UV beamer projection (xyz resolution 50 uµm ). In 30 minutes, post- printing (B), feeders were two times ultrasonic cleaned in 96% ethanol and flushed with 96% ethanol before they were dried with compressed air and post-cured for 10 minutes in a UV box (Nextdent LC-3DPrint Box, UV-A 108 en UV-Blue 108).


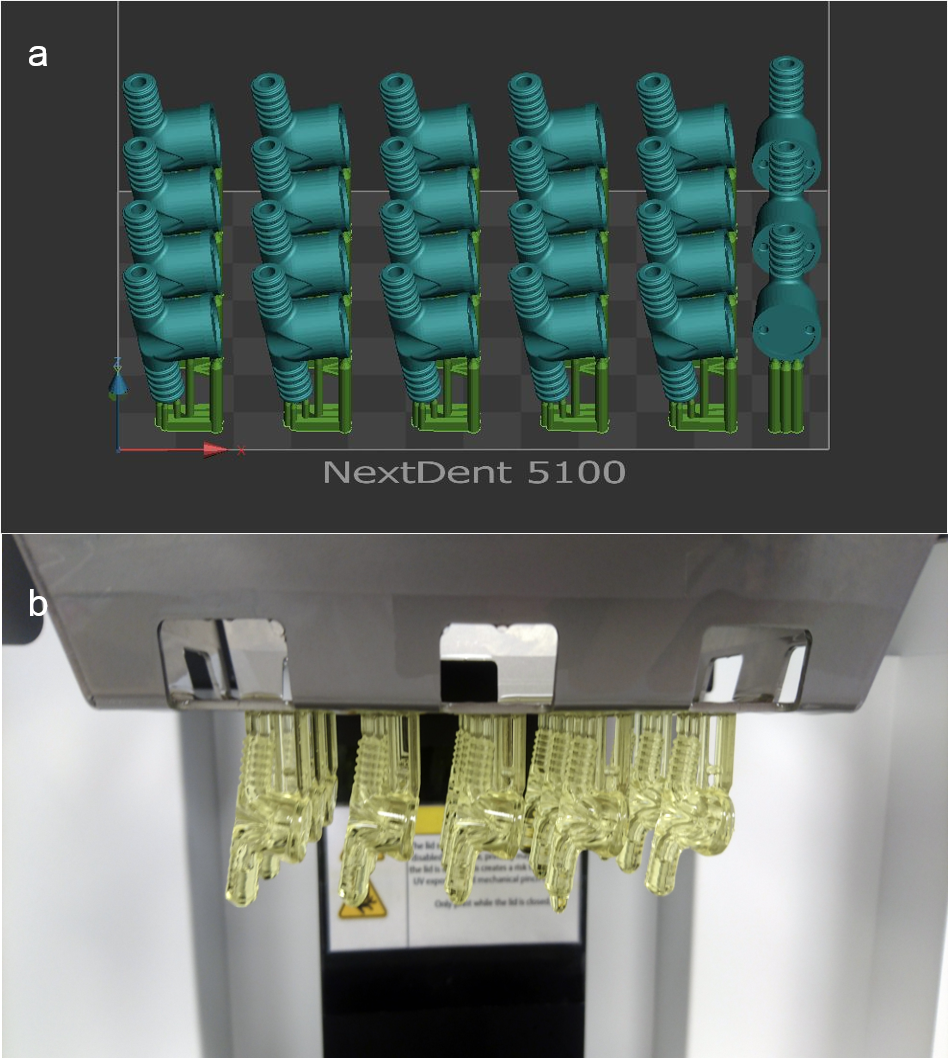

Supplement: Supplementary file 3 — Additional file 3: Figure S1. DLP nano-feeder production by Nextdent 5100. DLP feeders were designed using 3DSprint software from 3Dsystems. A total of 23 nano-feeders were printed in 1 h by the Nextdent 5100 Beta printer by the Radboudumc 3D lab [22]. Nextdent SG (surgical guide) was used as resin, no support material was used. Each layer of printed liquid resin was hardened by UV beamer projection (xyz resolution 50 µm). In 30 minutes, post printing (B), feeders were two times ultrasonic cleaned in 96% ethanol and flushed with 96% ethanol before they were dried with compressed air and post-cured for 10 min in a UV box (Nextdent LC-3DPrint Box, UV-A 108 en UV-Blue 108). [file 13071_2020_4269_MOESM3_ESM.docx]
