# Supplementary material for: A mosquito feeding assay to examine Plasmodium transmission to mosquitoes using small blood volumes in 3D printed nano-feeders
Source: Parasit Vectors. 2020 Aug 8;13:401. doi: 10.1186/s13071-020-04269-x (PMC7414548; doi:10.1186/s13071-020-04269-x)

**Additional file 4: Figure S2.** Feeder temperature of glass mini-feeders and 3D MJ-printed nano-feeders. Two probes were used to simultaneously measure the temperature of the first (P1) and last (P2) feeder in a row of four. At 0 s, the water bath was turned on (dotted line, temperature set at 39 °C) and the blood meal was injected in the feeder cavity of glass mini-feeders (circles) and nano-feeders (triangles). Both plastic and glass feeders reached a stable temperature quickly within 4.5 min after attaching to the water bath. Plastic feeders remained at a stable temperature on average 1.8 °C lower (mean = 35.9 °C) than glass feeders (mean = 37.7 °C).


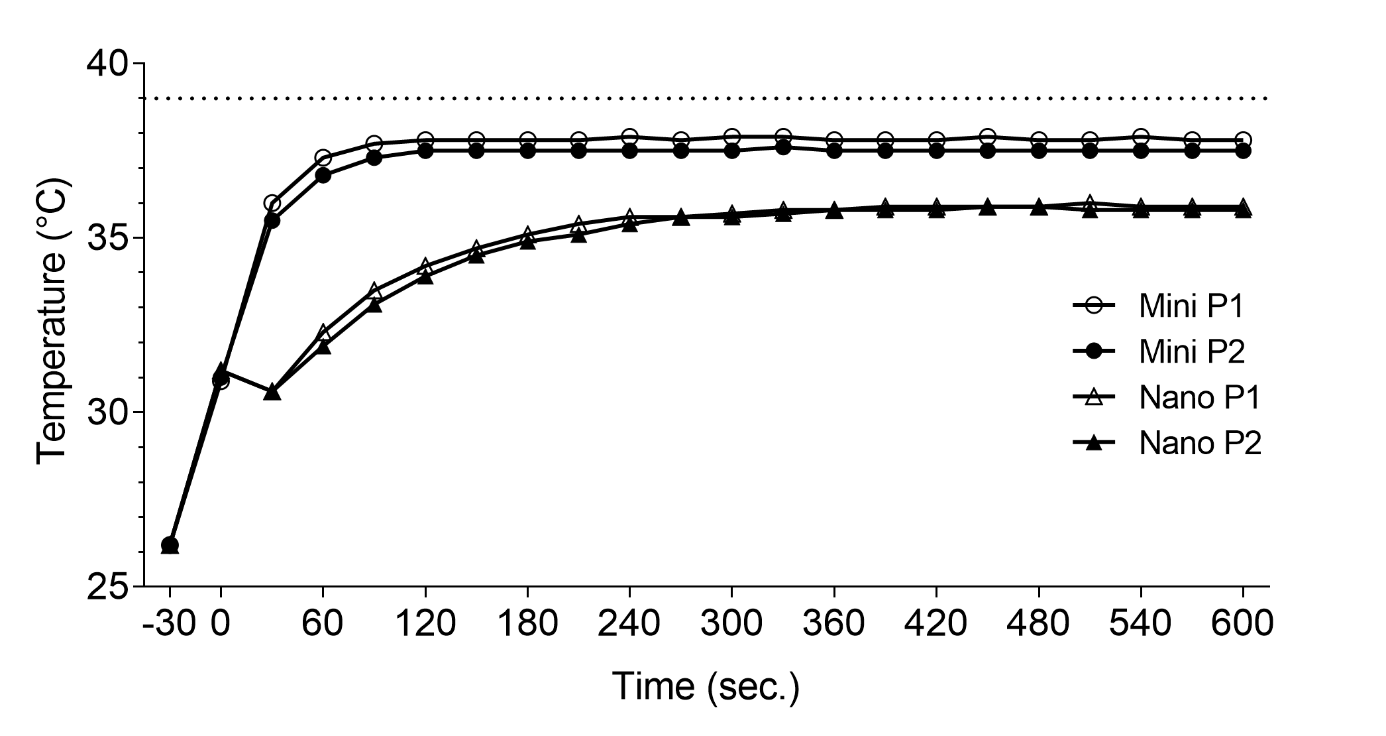

Supplement: Supplementary file 4 — Additional file 4: Figure S2. Feeder temperature of glass mini-feeders and 3D MJ-printed nano-feeders. Two probes were used to simultaneously measure the temperature of the first (P1) and last (P2) feeder in a row of four. At 0 s, the water bath was turned on (dotted line, temperature set at 39 °C) and the blood-meal was injected in the feeder cavity of glass mini-feeders (circles) and nano-feeders (triangles). Both plastic and glass feeders reached a stable temperature quickly within 4.5 min after attaching to the water bath. Plastic feeders remained at a stable temperature on average 1.8 °C lower (mean = 35.9 °C) than glass feeders (mean = 37.7 °C). [file 13071_2020_4269_MOESM4_ESM.docx]
