# Supplementary material for: A mosquito feeding assay to examine Plasmodium transmission to mosquitoes using small blood volumes in 3D printed nano-feeders
Source: Parasit Vectors. 2020 Aug 8;13:401. doi: 10.1186/s13071-020-04269-x (PMC7414548; doi:10.1186/s13071-020-04269-x)

**Additional file 5: Figure S3.** Mosquito blood-meal size determined by eye. Mosquitoes were selected by eye and determined unfed (UF), partially blood-fed (PBF) or fully blood-fed (FBF) (from left to right).


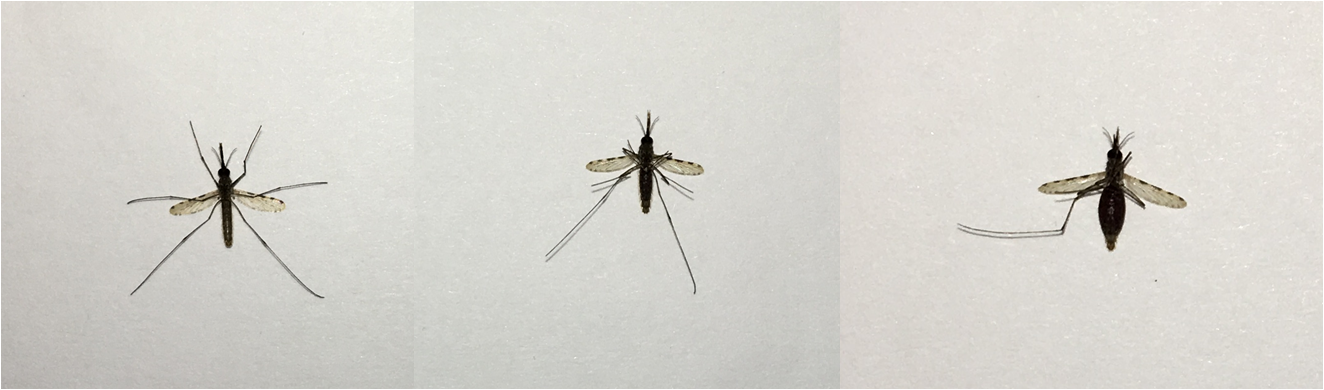

Supplement: Supplementary file 5 — Additional file 5: Figure S3. Mosquito blood-meal size determined by eye. Mosquitoes were selected by eye and determined unfed (UF), partially blood-fed (PBF) or fully blood-fed (FBF) (from left to right). [file 13071_2020_4269_MOESM5_ESM.docx]
