# Supplementary material for: A mosquito feeding assay to examine Plasmodium transmission to mosquitoes using small blood volumes in 3D printed nano-feeders
Source: Parasit Vectors. 2020 Aug 8;13:401. doi: 10.1186/s13071-020-04269-x (PMC7414548; doi:10.1186/s13071-020-04269-x)

**Additional file 6: Figure S4.** Mosquito feeding performance on 3D MJ-printed nano-feeders. In three independent experiments with three different blood donors, plastic cages with 5, 10, 15, 20 or 50 mosquitoes were fed on a 60 µl blood meal to determine the feeding performance on the nano-feeder. Data from independent experiments were pooled per group for analysis; pie charts present the mean feeding rate in percentages of fully- (FBF), partially- (PBF) or unfed (UF) mosquitoes. In all conditions a number of mosquitoes were PBF or UF. No blood material in the feeder cavity was left for cups with 20 or 50 mosquitoes. For 10 mosquitoes per feeder, all visually FBF mosquitoes had a blood meal > 2.5 µl, with a mean volume of 3.52 µl (range 2.6–4.0, *n* = 12) and feeding prevalence of 65% FBF.


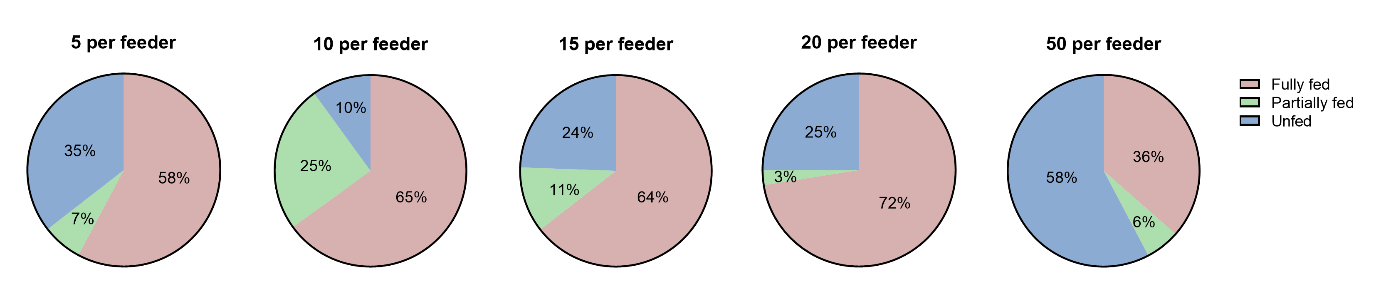

Supplement: Supplementary file 6 — Additional file 6: Figure S4. Mosquito feeding performance on 3D MJ-printed nano-feeders. In three independent experiments with three different blood donors, plastic cages with 5, 10, 15, 20 or 50 mosquitoes were fed on a 60 µl blood-meal to determine the feeding performance on the nano-feeder. Data from independent experiments were pooled per group for analysis; pie charts present the mean feeding rate in percentages of fully- (FBF), partially- (PBF) or unfed (UF) mosquitoes. In all conditions a number of mosquitoes were PBF or UF. No blood material in the feeder cavity was left for cups with 20 or 50 mosquitoes. For 10 mosquitoes per feeder, all visually FBF mosquitoes had a blood-meal > 2.5 µl, with a mean volume of 3.52 µl (range 2.6–4.0, n = 12) and feeding prevalence of 65% FBF. [file 13071_2020_4269_MOESM6_ESM.docx]
