# Supplementary material for: A mosquito feeding assay to examine Plasmodium transmission to mosquitoes using small blood volumes in 3D printed nano-feeders
Source: Parasit Vectors. 2020 Aug 8;13:401. doi: 10.1186/s13071-020-04269-x (PMC7414548; doi:10.1186/s13071-020-04269-x)
Supplement: Supplementary file 7 — Additional file 7: Table S1. Mosquito feeding rates after protocol optimization. To increase mosquito feeding rates the original nano-feeder protocol was optimised by (i) extension of the starvation period prior to feed (+ 12 h), (ii) stimulation of mosquitoes prior to feeding by placing a culture flask (canted neck 25 cm2) filled with warm water for 1 minute on top of cup, and (iii) plastic cages were changed to small paper cups to reduce distance between the mosquitoes and the feeder. [file 13071_2020_4269_MOESM7_ESM.docx]

**Additional file 7: Table S1.** Mosquito feeding rates after protocol optimization. To increase mosquito feeding rates the original nano-feeder protocol was optimised by (i) extension of the starvation period prior to feed (+ 12 h), (ii) stimulation of mosquitoes prior to feeding by placing a culture flask (canted neck 25 cm^2^) filled with warm water for 1 minute on top of cup, and (iii) plastic cages were changed to small paper cups to reduce distance between the mosquitoes and the feeder.

|  | **Nano-feeder mosquito feeding rates**  **(%[n/N])** | |
| --- | --- | --- |
|  | **Original protocol** | **Optimized protocol** |
| **1.** | 80% (8/10) | 90% (9/10) |
| **2.** | 70% (7/10) | 88.9% (8/9) |
| **3.** | 100% (10/10) | 90% (9/10) |
| **4.** | 100% (10/10) | 100% (10/10) |
| **5.** | 90 % (9/10) | 100% (10/10) |
| **6.** | 66% (6/9) | 90% (9/10) |
| **Range** | **66.7-100%** | **88.9-100%** |
| **Average feeding rate** | **84.5%** | **93.2** |
